# Coamplified Nanozyme Cocktails for Cascade Reaction-Driven Antioxidant Treatments

**DOI:** 10.1021/acsami.4c12511

**Published:** 2024-09-24

**Authors:** Tibor G. Halmagyi, Attila Voros, Szilard Saringer, Viktoria Hornok, Nora V. May, Gergely F. Samu, Imre Szenti, Adel Szerlauth, Zoltan Konya, Istvan Szilagyi

**Affiliations:** ‡ MTA-SZTE Momentum Biocolloids Research Group, Department of Physical Chemistry and Materials Science, Interdisciplinary Centre of Excellence, University of Szeged, 1 Rerrich Béla Tér, 6720 Szeged, Hungary; † Centre for Structural Sciences, HUN-REN Research Centre for Natural Sciences, 2 Magyar Tudósok Körútja, 1117 Budapest, Hungary; ∥ Department of Molecular and Analytical Chemistry, University of Szeged, 7 Dóm Tér, 6720 Szeged, Hungary; § Department of Applied and Environmental Chemistry, University of Szeged, 1 Rerrich Béla Tér, 6720 Szeged, Hungary

**Keywords:** reactive oxygen species, nanomaterial, Prussian blue, zeolite, colloidal stability

## Abstract

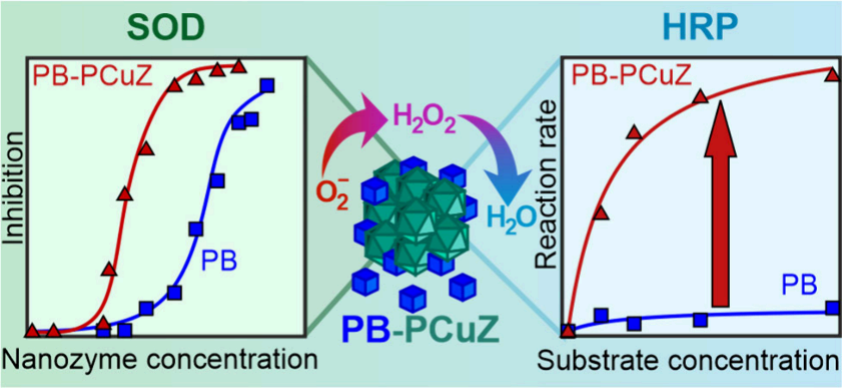

Antioxidant nanozymes are powerful tools to combat oxidative stress, which can be further improved by applying nanozyme mixtures of multiple enzymatic function. Here, cocktails of Prussian blue (PB) nanocubes and copper(II) exchanged ZSM-5 zeolites (CuZ) with enhanced reactive oxygen species (ROS) scavenging activity were developed. Surface functionalization of the particles was performed using polymers to obtain stable colloids, i.e., resistant to aggregation, under a wide range of experimental conditions. The nanozyme cocktails possessed advanced antioxidant properties with multiple enzyme-like functions, catalyzing the decomposition of ROS in cascade reactions. The activity of the mixture far exceeded that of the individual particles, particularly in the peroxidase assay, where an improvement of more than an order of magnitude was observed, pointing to coamplification of the enzymatic activity. In addition, it was revealed that the copper(II) site in the CuZ plays an important role in the decomposition of both superoxide radicals and hydrogen peroxide, as it directly catalyzes the former reaction and acts as cocatalyst in the latter process by boosting the peroxidase activity of the PB nanozyme. The results give important insights into the design of synergistic particle mixtures for the broad-spectrum scavenging of ROS to develop efficient tools for antioxidant treatments in both medical therapies and industrial manufacturing processes.

## 1 Introduction

Enzymes are at the forefront of the rapidly growing technological field of biocatalysis offering efficient and selective catalytic routes for chemical reactions at mild conditions in biomedical, environmental, and industrial applications.^1−5^ A drawback of these materials is the necessity of precise temperature, pH, and ionic strength control, which often prevents the use of native enzymes as biocatalysts.^6^ Nanozymes, i.e., enzyme mimicking nanoparticles,^7−9^ offer an alternative solution for such limitations. Their cost-effective preparation, storage, and chemical stability are the main advantages compared to their biological counterparts. In addition, nanozymes are generally less sensitive to changes in the experimental conditions.^10^ Precisely engineered nanostructures can show comparable activity or even outperform native enzymes.^11,12^ Furthermore, nanozyme composites can mimic enzymatic cascades, i.e., carry out a series of enzyme-catalyzed processes, where the product of one reaction is the substrate for the next one.^13^ Such a multiple activity effect is beneficial for single nanozyme particles as local substrate concentrations for successive reactions remain high, resulting in an increased efficiency.^14,15^

Regarding antioxidant enzyme mimics, the most important complex reaction is the decomposition of superoxide radical anions to molecular oxygen and water. The biological enzyme superoxide dismutase (SOD)^16^ catalyzes the disproportionation of superoxide radicals into oxygen and hydrogen peroxide.^17^ Since the latter product is also a reactive oxygen species (ROS), it is then further broken down into water and oxygen by catalase (CAT) or peroxidase (POD) enzymes. This tandem enzymatic process is crucial for protecting cells from oxidative stress induced by elevated ROS levels.^18^ Therefore, researchers are actively developing SOD, CAT, and POD-mimicking nanozymes, as they represent exciting frontiers in various fields, where artificial catalysts can be designed to replicate and even enhance the protective functions of natural enzymes.^19^

Accordingly, a wide range of antioxidant nanozymes of SOD, CAT, and POD-like activities have been developed for tandem catalytic applications recently, including clays,^20^ metal oxides,^21^ carbon derivatives,^22^ and other nanoparticles.^23,24^ Among the latter ones, Prussian blue (PB), a coordination polymer composed of cyanide ligands bridging iron cations of mixed valence,^25,26^ proved to be a potential antioxidant nanozyme due to its ability to scavenge ROS via multiple enzymatic activities such as SOD, CAT, and POD.^27,28^ Synthesis of PB of advantageous structural features attracts widespread interest in the research communities.^29−34^

The use of highly ordered and porous materials such as zeolites is beneficial in reactions requiring size selectivity and specificity. These catalysts can withstand high temperatures and varying reaction conditions.^35^ Structural modification methods such as aqueous ion exchange also allow the incorporation of guest materials into the host aluminosilicate framework, combining the individual catalytic activity of host and guest.^36^ Despite these promising properties of zeolites and the fact that similarly structured materials have been considered for potential applications as nanozymes,^37^ research on zeolite-based nanozyme systems is still a relatively unexplored domain.

Apart from single-nanozyme systems, more elaborate architectures composed of multiple enzymatically active materials have also been prepared to further enhance their ROS scavenging ability. For example, copper–ceria composites exhibited remarkable POD-like activity due to the interaction between the coordinated metal and the carrier particle, and the composite was successfully utilized in antibacterial applications.^38^ A gold–copper oxide heterostructure was reported as a multienzyme-mimicking compound of complete antioxidant capacity to scavenge ROS, as confirmed in cellular and animal tests.^39^ Platinum–copper nanoalloys inhibited cell death and neuron-to-neuron transmission by scavenging ROS in primary neuron cultures.^40^ While these complex materials were potent antioxidants, their preparation is complicated and similar broad-spectrum ROS decomposition may be achieved by simply mixing individual nanozymes of different enzyme-like operations.^10^ Nevertheless, there is a lack of literature reports in which nanozyme cocktails of multiple functions were developed for cascade ROS neutralization. In addition, despite the fact that antioxidant nanozymes are mostly used in liquid media, the colloidal properties of such systems were not assessed and optimized.

The objective of this study is therefore to develop mixed nanozyme systems to mimic antioxidant cascade reactions. While individual antioxidant materials were frequently reported in the past, there is a lack of comprehensive studies on the development of colloid mixtures of different particles for efficient ROS scavenge. For this, PB and copper(II)-exchanged ZSM-5 zeolite particles (CuZ) were used. CuZ has proven its catalytic activity in various reactions;^41−45^ however, no reliable studies on CuZ-based antioxidant systems exist. Mixtures of PB and CuZ of different mass ratios were characterized from structural and colloidal aspects using various microscopy, scattering, and spectroscopy techniques. The influence of the composition, structure, and dispersion features on the enzyme-like activity of the cocktails obtained was systematically evaluated.

## 2 Materials and Methods

### 2.1 Materials

Copper(II)-acetate (≥98%), hydrogen peroxide (∼31 wt %), and poly(diallyldimethylammonium chloride) (PDADMAC, average *M*_w_ ∼ 225 kDa, 20 wt %) were procured from Sigma-Aldrich. Ammonium-ZSM-5 zeolite (23:1 Si:Al ratio) was obtained from Thermo Scientific. Guaiacol (99%), hydrogen chloride (37 wt %), nitro blue tetrazolium chloride monohydrate (NBT, ≥98%), polyvinylpyrrolidone (PVP, *M*_w_ ∼ 50 kDa), potassium hexacyanoferrate(III) (99%), sodium chloride (NaCl, 99.8%), sodium hydroxide (98.5%), sodium phosphate (monobasic, reagent grade), and sodium phosphate (dibasic, reagent grade) were purchased from VWR. Ultrapure water was produced by an Adrona water purification system.

### 2.2 Synthesis of PB Nanocubes

The synthesis of PVP-templated PB nanocubes was adapted from literature.^30^ In brief, 9.0 g of PVP and 339.3 mg of potassium hexacyanoferrate(III) were added to a 10 mM hydrogen chloride solution. The resulting mixture was stirred at 80 °C for 20 h. Over this time, the solution slowly turned green and then deep blue, indicating the formation of the PB material. The product was centrifuged and washed several times with ultrapure water set to pH 4 with hydrogen chloride and then diluted to form a 2000 ppm stock solution. The samples were stored at 4 °C and remained stable for several months. The successful formation of PB was confirmed with Raman spectroscopy and UV–visible spectrophotometry, as detailed later.

### 2.3 Synthesis of Copper(II)-Containing ZSM-5 Zeolite (CuZ)

An aqueous ion-exchange method was adapted from the literature to form copper(II)-containing zeolites (CuZ).^46^ In a typical synthesis, a given amount of ammonium-ZSM-5 was treated with 100 mL/g (with respect to zeolite weight) copper(II) acetate solution (5 mM) for 24 h under stirring. The solution was then centrifuged, and the same treatment was repeated. Then, the product was centrifuged and washed several times with ultrapure water, dried at 60 °C, and annealed at 550 °C to remove the residual ammonium ions. The final CuZ product was stored as a dried powder at 4 °C. The structure and the composition of CuZ were investigated with X-ray photoelectron (XPS) and electron paramagnetic resonance (EPR) spectroscopies; see description in the Supporting Information.

### 2.4 Assembly of the PB–PCuZ Mixtures

**Figure 1:**
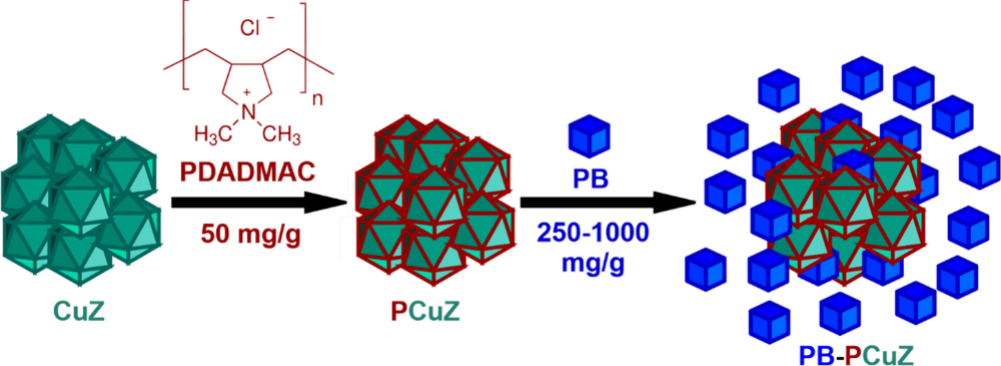
Schematic illustration of the PB–PCuZ fabrication method.

In a typical procedure (Figure 1), a CuZ dispersion (pH 4) was prepared by thorough ultrasonic treatment to minimize particle size, and then, 50 mg/g (with respect to CuZ content) PDADMAC was introduced. After 30 min, 250 or 1000 mg/g PB was added, and the mixtures (denoted as PB–PCuZ (1:4) and PB–PCuZ (1:1), respectively) were allowed to equilibrate for at least 1 h before use. The samples were stored at 4 °C. While sedimentation of the dispersions occurs, samples could be redispersed and homogenized using ultrasonic treatment.

### 2.5 Transmission Electron Microscopy (TEM)

TEM images of both individual nanozymes and the PB–PCuZ composites were recorded by a Jeol JEM-1400Plus type instrument (Japan) at a 120 keV accelerating voltage. Prior to measurements, 10 μL aliquots of the sample dispersions were dropped onto carbon-coated Formvar foil 200 mesh copper grids and left drying. The size distribution of PB and CuZ particles was determined with the automatic particle analysis function of the ImageJ software.

### 2.6 Scanning Electron Microscopy Coupled Energy Dispersive X-ray Spectroscopy (SEM-EDX)

The elemental composition was determined using EDX built in a Thermo-Fisher Scientific Apreo C SEM operating at a 40 nA current and 20 kV acceleration voltage.

### 2.7 Raman Spectroscopy

Raman spectra of the PB–PCuZ mixtures as well as the individual components were recorded by using a Bruker Senterra II Raman microscope equipped with a light source of 532 nm and 25 mW laser power. The reported data were obtained by averaging 32 spectra with an exposition time of 4 s.

### 2.8 EPR Spectroscopy

All EPR spectra were recorded with a BRUKER EleXsys E500 spectrometer (microwave frequency 9.54 GHz, microwave power 13 mW, modulation amplitude 5 G, and modulation frequency 100 kHz). For the frozen solution spectra, 0.20 mL samples were mixed with 0.05 mL of methanol (to avoid crystallization of the water) and transferred into quartz EPR tubes, and the data were recorded in Dewar containing liquid nitrogen (77 K). The anisotropic EPR spectra were analyzed by taking into account axial **g**-tensor (*g*_⊥_, *g*_∥_) and copper hyperfine tensor (*A*_⊥_^Cu^, *A*_∥_^Cu^). Orientation dependent line width parameters (α, β, and γ) were used to fit the line widths through eq 1:
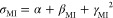
1where MI denotes the magnetic quantum number of copper(II) ion. Since a natural copper(II) chloride was used for the measurements, the spectra were calculated by the summation of spectra of ^63^Cu and ^65^Cu weighted by their natural abundances. The hyperfine and superhyperfine coupling constants and the relaxation parameters were obtained in field units (Gauss = 10^–4^ T).

### 2.9 XPS Analysis

A 150 W Al Kα X-ray source (*h*ν = 1486.6 eV) was used to perform the XPS measurements with a SPECS instrument equipped with a PHOIBOS 150 MCD 9 hemispherical analyzer, which operated in fixed transmission mode with 40 eV pass energy for acquiring survey scans in the 0–1200 eV binding energy range. For the high-resolution scans, a 20 eV pass energy was used. Charge referencing was performed for the adventitious carbon C 1s peak (284.8 eV). For the spectrum evaluation, the CasaXPS commercial software package was used.

### 2.10 Dynamic Light Scattering (DLS)

DLS measurements were performed on a LS spectrometer (LS Instruments) in 3D modulated cross-correlation mode.^47^ The instrument is equipped with a 633 nm He–Ne laser of 120 mW maximum power. All of the measurements were carried out in backscattering mode at a scattering angle of 150°. The CORENN algorithm (developed by LS Instruments) was applied to fit the intensity autocorrelation functions for yielding the translational diffusion constant of the particles used to calculate the hydrodynamic radius with the Stokes–Einstein equation.^48^

### 2.11 Electrophoretic Light Scattering (ELS)

Electrophoretic mobilities were determined with ELS by using a Litesizer 500 device (Anton Paar). The instrument is equipped with a 40 mW laser. Polycarbonate-based “omega” cuvettes equipped with gold-coated electrodes (Anton Paar) served as sample holders. All ELS measurements were performed at a 10 mM sodium chloride concentration, unless otherwise stated, to adjust the ionic strength. The samples were equilibrated at 25 °C during both the ELS and DLS experiments.

### 2.12 SOD Assay

The SOD-like activity of the nanozyme systems was determined with a modified Fridovich assay.^49^ Accordingly, a reaction mixture was prepared such that it contained 200 μM xanthine, 100 μM NBT, and a varying amount of nanozyme, usually with an active component content between 0 and 100 ppm. All stock solutions, except the nanozyme, were prepared in 10 mM phosphate buffer (pH 6.9). Upon addition of 300 ppm of xanthine oxidase, the enzyme catalyzes the oxidation of xanthine to uric acid, producing superoxide radical anions that, in the absence of a superoxide scavenger, react with NBT to produce purple-colored formazan. The formation of this product can be followed by monitoring the absorbance at 565 nm wavelength (with a GENESYS 10S spectrophotometer) for 6 min. The inhibition (*I*) activity of a given nanozyme amount can be then defined as follows:
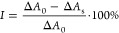
2where Δ*A*_0_ is the change in absorbance over 6 min without nanozyme, and Δ*A*_s_ is the absorbance change in a sample with a given nanozyme concentration. An inhibition curve can be constructed by plotting the data calculated with eq 2 as a function of the nanozyme concentration. The quantitative characteristic of a SOD-mimicking nanozyme on the inhibition plot is the IC_50_ value, i.e., the nanozyme concentration that causes 50% inhibition. This was determined by a nonlinear fit of the inhibition–concentration plots of the following form:
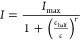
3where the three parameters are *I*_max_ (the maximum inhibition), *c*_half_ (nanozyme concentration at half-maximum, i.e., equal to the IC_50_ value if *I*_max_ is 100%), *r* is the slope of the curve, and *c* is the nanozyme concentration. The error of this method is about 10%.

### 2.13 POD Assay

To ascertain the POD-like activity of the nanozymes studied, a method utilizing guaiacol as substrate was adapted from the literature.^50^ In a typical experiment, a series of reaction mixtures was prepared, each containing a given concentration of nanozyme (10 or 40 ppm, fixed for each measurement series), a varying amount of guaiacol (0–40 mM), and 2.7 mM hydrogen peroxide added at the start of the measurement. Each experiment was conducted in a 50 mM phosphate buffer (pH 6.9). Immediately upon addition of hydrogen peroxide and in the presence of a nanozyme catalyst, the guaiacol is oxidized to tetraguaiacol, which has a characteristic brown color. This reaction can be monitored by measuring the time-dependent increase in the absorbance of the solution at a wavelength of 470 nm. By plotting the recorded absorbance as a function of time, the reaction rate (*v*) can be calculated using eq 4 adapted from the Beer–Lambert law:
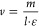
4where *m* is the slope of the absorbance versus time plot, *l* is the length of the light path through the sample (1 cm), and ε is the molar absorption coefficient of the tetraguaiacol product (26.6 1/(mM cm)). The reaction rate was then plotted as a function of the guaiacol concentration, and a nonlinear fit was performed on the data points using the Michaelis–Menten equation:^51^
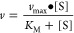
5where *v*_max_ is the theoretical maximum reaction rate, [S] is the concentration of the guaiacol substrate, and *K*_M_ is the so-called Michaelis constant indicating [S] value, at which the reaction rate is half of *v*_max_. The catalytic turnover rate (*k*_cat_) can be calculated from *v*_max_:
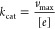
6where [*e*] is the concentration of active sites. Nevertheless, note that using *k*_cat_ for characterizing the activity of nanozymes can be misleading as ordinarily either one particle or one metal atom is considered an active site.^52^ The former approach, also used in this article, can overestimate, while the latter approach can underestimate catalytic turnover rates.^8^

Another widely used substrate for the determination of POD-like activity is 3,3′,5,5′-tetramethylbenzidine (TMB). An assay adapted from literature was used to characterize the POD-activity of the nanozymes with TMB and to compare the results with the above-discussed guaiacol test.^10^ In the presence of H_2_O_2_ and a POD-mimicking material, the colorless TMB is oxidized to yield a blue product with a characteristic absorbance peak at 652 nm. In a typical test, a reaction mixture containing 40 ppm active component (PB in the case of PB and PB–PCuZ, CuZ otherwise) and 0.5 mM TMB (from a 10 mM stock solution in a DMSO:water 1:1 mixture) was prepared in 10 mM phosphate buffer (pH 6.9). To this system, 15 mM H_2_O_2_ was added, and after 10 min reaction time, the absorbance of the mixtures was measured between 400 and 800 nm. As PB and the PB–PCuZ mixtures absorb heavily around 652 nm, each sample was measured against a blank mixture containing 40 ppm of nanozyme and 15 mM H_2_O_2_ in 10 mM phosphate buffer. Relative activities were calculated from the absorbance values of the nanozyme mixtures at 652 nm, with the highest value selected as 100%. The average error of the POD assays was 10%.

## 3 Results and Discussion

### 3.1 Colloidal Characterization of the PB–PCuZ Systems

**Figure 2:**
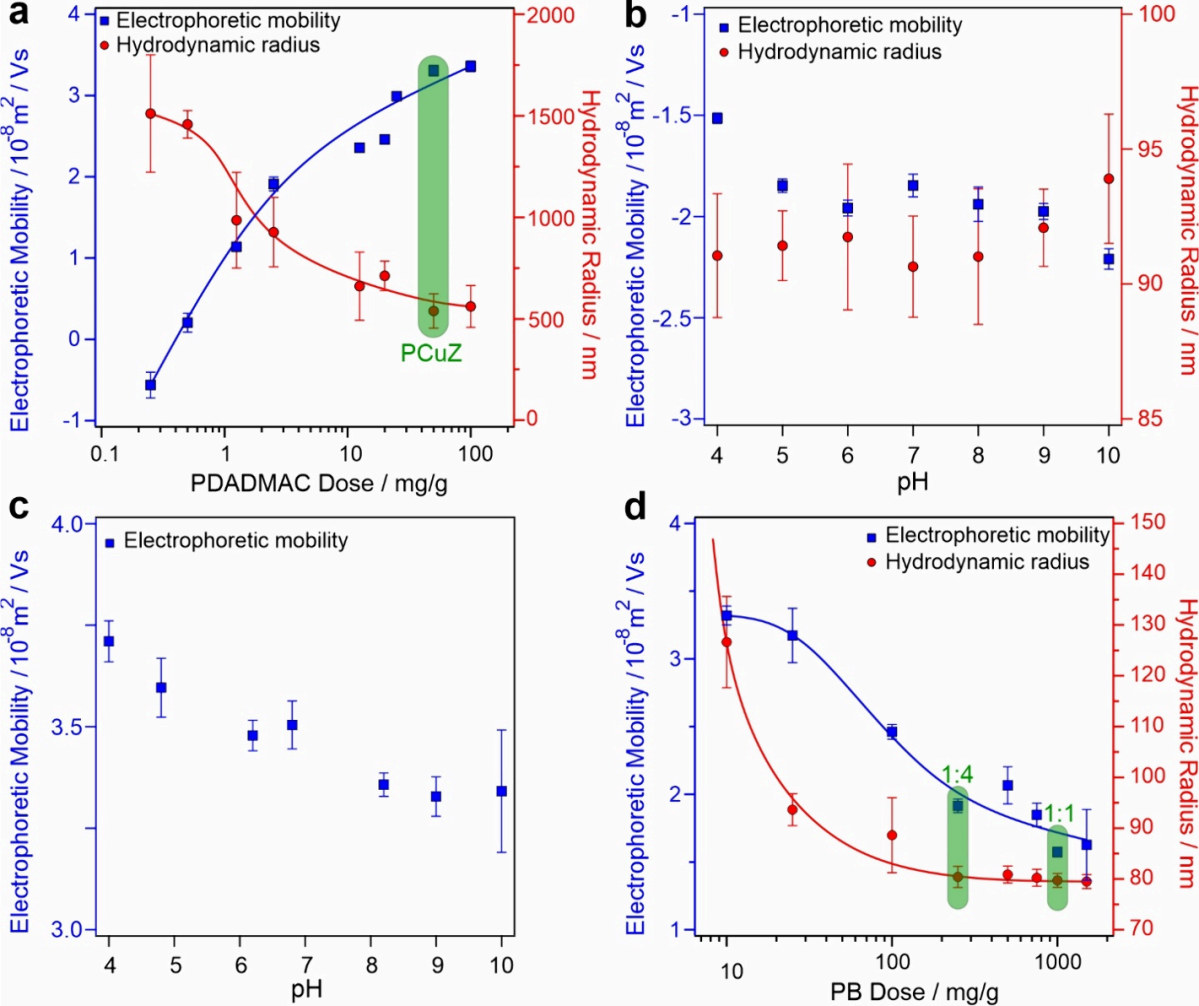
Electrophoretic mobilities and hydrodynamic radii data for CuZ particles as a function of the PDADMAC dose (a), PB nanocubes (b), and PCuZ (with 50 mg/g PDADMAC) (c) versus the pH as well as PCuZ particles as a function of the PB dose (d). Dose represents mg dosing material (PDADMAC or PB) per gram of CuZ. The green areas indicate the doses applied in future experiments. The lines are eye guides.

Both CuZ and PB were found to be negatively charged at the experimental conditions applied, and thus, the surface of CuZ was functionalized with oppositely charged PDADMAC polyelectrolyte to induce possible heteroaggregation between CuZ and PB via electrostatic forces. Accordingly, CuZ suspension of 40 ppm concentration was treated with varying concentrations of PDADMAC, and the electrophoretic mobility and hydrodynamic radius and of the particles were determined with ELS and DLS, respectively (Figure 2a).

The initially negative electrophoretic mobility value of the CuZ rapidly reaches the isoelectric point (IEP, the dose where the net surface charge is zero) around a 0.6 mg/g polymer dose. Further increasing the PDADMAC level leads to sign reversal of the electrophoretic mobilities, and the so-called overcharging phenomenon takes place. Above 50 mg/g, the increase in the electrophoretic mobility stops as the so-called adsorption saturation plateau (ASP) is reached. In other words, at the onset of this plateau, at 50 mg/g PDADMAC dose (indicated with a green area in Figure 2a), the surface of the CuZ is saturated with the adsorbed polyelectrolyte.

The hydrodynamic radius of particles was monitored under the same conditions as in the mobility study, i.e., the same PDADMAC concentration range (Figure 2a). The radii decreased to 500 nm at the highest polymer doses. Comparison of tendencies in the particle mobility and radius revealed that higher charge leads to lower size indicating that electrostatic repulsion between the like-charged particles prevents aggregation processes, similar to other polyelectrolyte coated particle systems.^10,15^ As the goal was to obtain positively charged particles with the smallest possible size, a 50 mg/g PDADMAC dose was selected (denoted as PCuZ particles) to induce possible heteroaggregation in the PB–PCuZ mixtures via electrostatic surface forces.

To further confirm the charge balance and to assess the colloidal behavior of the PB and PCuZ nanozymes as a function of pH, DLS and ELS measurements were carried out (Figure 2b,c). The charge and size of PB nanocubes do not depend significantly on the pH (Figure 2b), similar to other types of PB particles reported earlier.^28^ In addition, the polydispersity index of the material was very low (0.07), indicating narrow particle size distribution. The PB nanozyme retains its size even in concentrated electrolytes at pH 4 (Figure S1). Slow dissolution of the material can be observed at pH 10 in a much larger time frame than the experiments performed in this work (Figure S2); however, this did not take place at lower pH values. On the other hand, only about 10% decrease in the electrophoretic mobility values (Figure 2c) from pH 4 to 10 indicates slight pH-dependent surface charge for the PCuZ particles. This result indicates that pH does not play a significant role in PB–PCuZ formation, as both components exhibit pH-independent surface charge in the pH range used. Note that the PCuZ particles were too polydisperse in size to apply DLS in the entire pH range studied owing to the presence of aggregates, which somewhat disappeared upon PDADMAC treatment at pH 4.

The charge and size features in the PB–PCuZ mixtures were investigated by treating the PDADMAC-coated CuZ with increasing doses of PB. The changes in the electrophoretic mobility and hydrodynamic radius were monitored (Figure 2d). Due to the sixth power dependence of the scattered intensity on the size of the scattering particle, one would ordinarily expect the larger PCuZ to be over-represented in the hydrodynamic radius values.^53^ However, as indicated by the laser intensity-scaled count rates (Table S1 in the Supporting Information) light scatters more than 20 times more intensely from PB nanocubes than from PCuZ at the same mass concentration. Thus, even at very low PB doses the hydrodynamic radius values obtained from DLS measurements skew heavily toward the size of the PB particles. This result also indicates that a significant portion of PB particles is not attached to the oppositely charged PCuZ but rather dispersed individually in the dispersion. The trend in the electrophoretic mobility values is in line with the above observation as the data decrease once the positively charged PCuZ particles are dosed with the negatively charged PB due to partial charge neutralization upon adsorption and the contribution of the individual PB particles to the electrophoretic mobility values. However, a reversal in the sign of charge was not detected. To further characterize the PB–PCuZ mixtures, 1:4 and 1:1 PB:PCuZ mass ratios (indicated by a green area in Figure 2d) were selected for further studies, as a stable dispersion formed under these conditions. These mixtures are denoted as PB–PCuZ (1:4) and PB–PCuZ (1:1), respectively, in the following sections.

### 3.2 Characterization of Morphology and Structure

**Figure 3:**
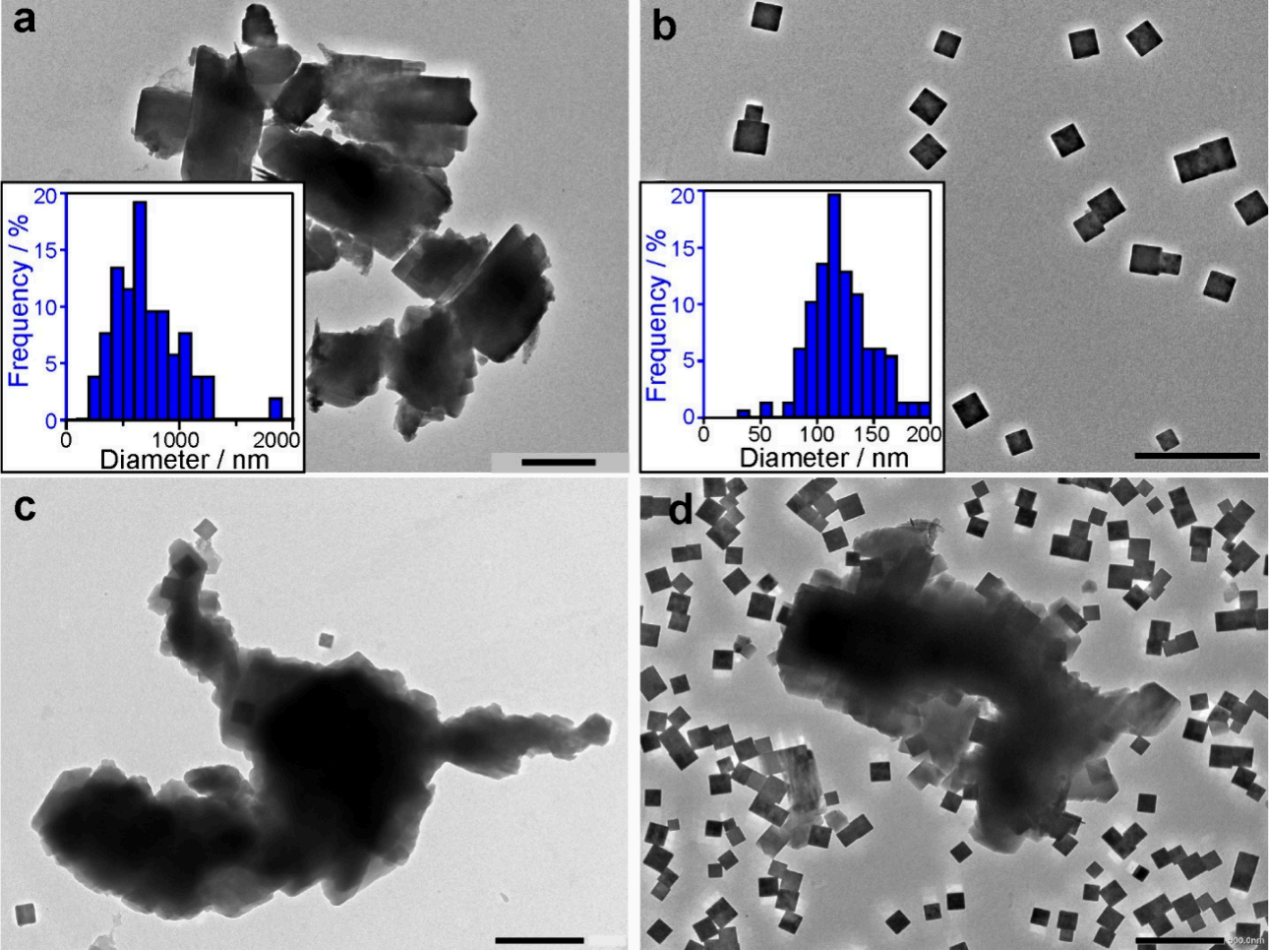
TEM images of CuZ (a), PB (b), PB–PCuZ (1:4) (c), and PB–PCuZ (1:1) (d) samples. The scale bars represent 500 nm. The insets in (a) and (b) represent the size distribution of CuZ and PB, respectively, determined with the ImageJ software.

Figure 3 shows the TEM images of CuZ, PB, PB–PCuZ (1:1), and PB–PCuZ (1:4) particles. Aggregation upon drying can be observed in the CuZ sample (Figure 3a), with clusters of several μm in diameter formed from the primary particles, which indicates the relatively high polydispersity and the irregular shape of the particles or aggregates. This is also reflected in the size distribution of the materials (Figure 3a, inset). The absence of small particles in the images implies that no other copper moieties were formed along with CuZ.^54^ The PB sample consists of cubes of approximately 100 nm in diameter (Figure 3b), with finite polydispersity (Figure 3b, inset). The individual PB nanozymes are well-dispersed, and the formation of large aggregates did not occur. Moreover, PB nanocubes are present both independently and adsorbed on the zeolite surface in the PB–PCuZ samples (Figure 2c,d). The expected heteroaggregation driven by attractive electrostatic interactions between the oppositely charged particles^55^ took place only partly in these systems. Accordingly, the PB–PCuZ (1:1) and PB–PCuZ (1:4) dispersions are mixtures of the two types of materials with only a fraction of PB adsorbed on the zeolite surface. Indeed, the electrophoretic mobility data discussed earlier (Figure 2d) confirm this observation, as it represents an average value for the mixed system.

The Raman spectra of the individual nanozymes as well as of the PB–PCuZ (1:1) composite were recorded and are shown in Figure S3 together with the peak assignment (Table S2). Both the CuZ and the PB spectra closely follow other literature data on similar materials.^56,57^ In the high-wavenumber region (Figure S3a), the 2095 cm^–1^ peak represents the Fe–CN–Fe stretch, and the peak at 2156 cm^–1^ stands for the C≡N stretching vibration in PB. In a comparison of the data obtained for PB–PCuZ (1:1) to the PB and PCuZ samples, no shifts in band locations were observed in this regime. The symmetrical stretching vibration at 380 cm^–1^ and the bending vibration at 295 cm^–1^ can be attributed to the T–O–T bonds, where T are the tetrahedrally coordinated central atoms, mainly silicon (Figure S3b). The peaks at 400–500 cm^–1^ (red box, Figure S3b) are the superposition of 3 overlapping vibrations, namely, Si–O–Si stretching, O–Si–O(Al) bending, and Si–O bending, while the peak at 790 cm^–1^ is assigned to the Si–O–Si symmetrical stretching vibration. The peak at 279 cm^–1^ corresponds to the Fe–CN–Fe bending vibration, while the wide peak at 450–620 cm^–1^ (green boxes, Figure S3b) is assigned to the Fe–C stretching vibration. The spectrum of the PB–PCuZ mixtures contains peaks identical to those of PB.

UV–visible spectra of the relevant nanozyme systems were recorded (Figure S4). The CuZ does not show the usual characteristic copper(II) peak at around 750 nm ^54^ as the approximately 1 atomic % copper-content is not sufficiently high for detection. The sample only presents near-constant absorbance that slightly increases toward lower wavelengths. In contrast, the PB dispersions show the characteristic absorption spectrum reported for such materials,^58^ with a broad peak around 720 nm and another one toward the UV region.

SEM-EDX analysis was performed on the ZSM-5 precursor and CuZ and PB–PCuZ samples to determine the elemental makeup of these materials (Figure S5). EDX spectra were recorded from 0 to 10 keV energy on the ZSM-5 control sample (Figure S5a) and the nanozymes. All measured data contain the peaks frequently attributed to zeolites,^59^ while the copper-exchanged derivatives (Figure S5b–d) and PB-containing samples (Figure S5c and Figure S5d for PB–PCuZ (1:4) and (1:1), respectively) also bear the corresponding metal peaks. No other elements can be observed, indicating the purity of the compounds synthesized. The summary of the results obtained with EDX elemental analysis is presented in Figure S6. It was found that oxides dominate the chemical composition of all samples (Figure S6a). Upon ion exchange with copper(II) acetate and subsequent annealing, the nitrogen content (3.12 ± 0.05% in the original ammonium-ZSM-5) disappears, while copper is present in all subsequent samples (0.93 ± 0.02%, 0.25 ± 0.02%, and 0.11 ± 0.03% in CuZ, PB–PCuZ (1:4), and PB–PCuZ (1:1), respectively), as shown in Figure S6b. The amount of copper on the surface of CuZ determined with XPS (Figure S7) is in good agreement with the results from EDX, signaling that the copper(II) ion exchange progressed to the bulk of the zeolite material beyond the surface. The EDX results also reveal that both PB–PCuZ mixtures contain also iron (0.05 ± 0.03% and 0.22 ± 0.03% in the (1:4) and (1:1) sample, respectively), as expected from the previous results. The values indicate within the measurement error that the (1:1) mixture indeed contains four times more PB as the (1:4) sample. The carbon contents are significantly higher than that of the ZSM-5 and CuZ samples, owing to the presence of the PVP-coated PB nanocubes.

### 3.3 SOD Activity

**Figure 4:**
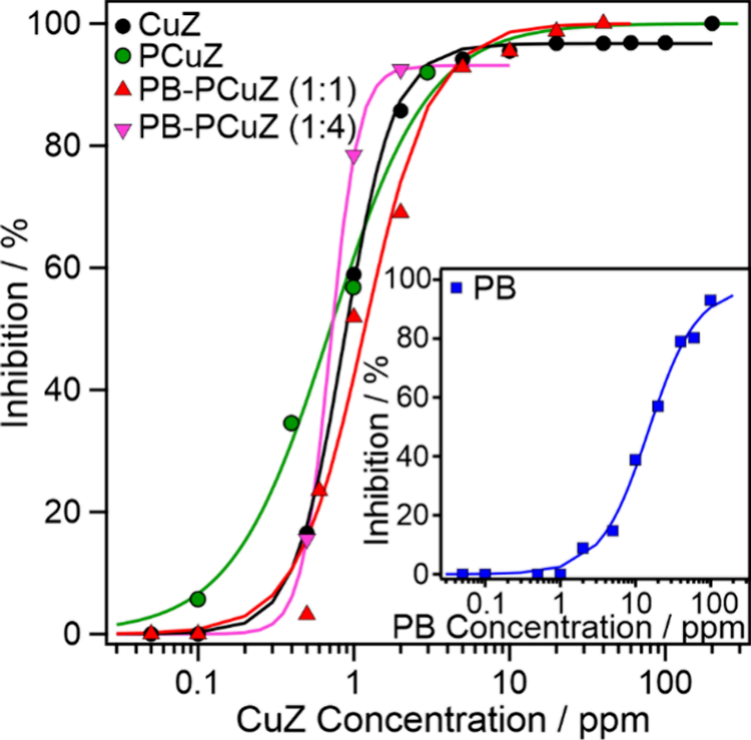
SOD-like activity of CuZ-containing materials (data for the pure PB systems are shown in the inset). Xanthine concentration was maintained at 0.2 mM for all experiments. The lines are the results of nonlinear fits following eq 3 and were used for IC_50_ calculations.

The SOD function of the nanozymes was investigated with a modified version of the Fridovich assay,^49^ wherein the ability of the nanozymes to scavenge the superoxide radicals from the oxidation of xanthine was assessed. The IC_50_ values were determined from a nonlinear fit on the inhibition versus nanozyme concentration data (see eq 3). The CuZ-containing samples exhibit almost identical activities and IC_50_ values (CuZ 0.90 ± 0.02 ppm, PCuZ 0.6 ± 0.1 ppm, PB–PCuZ (1:1) 1.2 ± 0.1 ppm, PB–PCuZ (1:4) 0.7 ± 0.2 ppm) regardless of the PB content (Figure 4), while the value for PB nanocubes is an order of magnitude higher at 15.0 ± 1.6 ppm (Figure 4, inset). Thus, CuZ possesses superior SOD-like activity compared to the PB nanocubes, and this activity is not affected by mixing with PB. It is likely that the superoxide radical product of the enzyme-catalyzed oxidation of xanthine penetrates into the zeolite structure, where its decomposition is catalyzed by the copper(II) centers. The inferior activity of the PB nanocubes compared to other similar materials^28^ can be explained by the PVP coating on the nanocube surface, which may block some active sites and hence limit the enzyme-like function.

### 3.4 POD Activity

**Figure 5:**
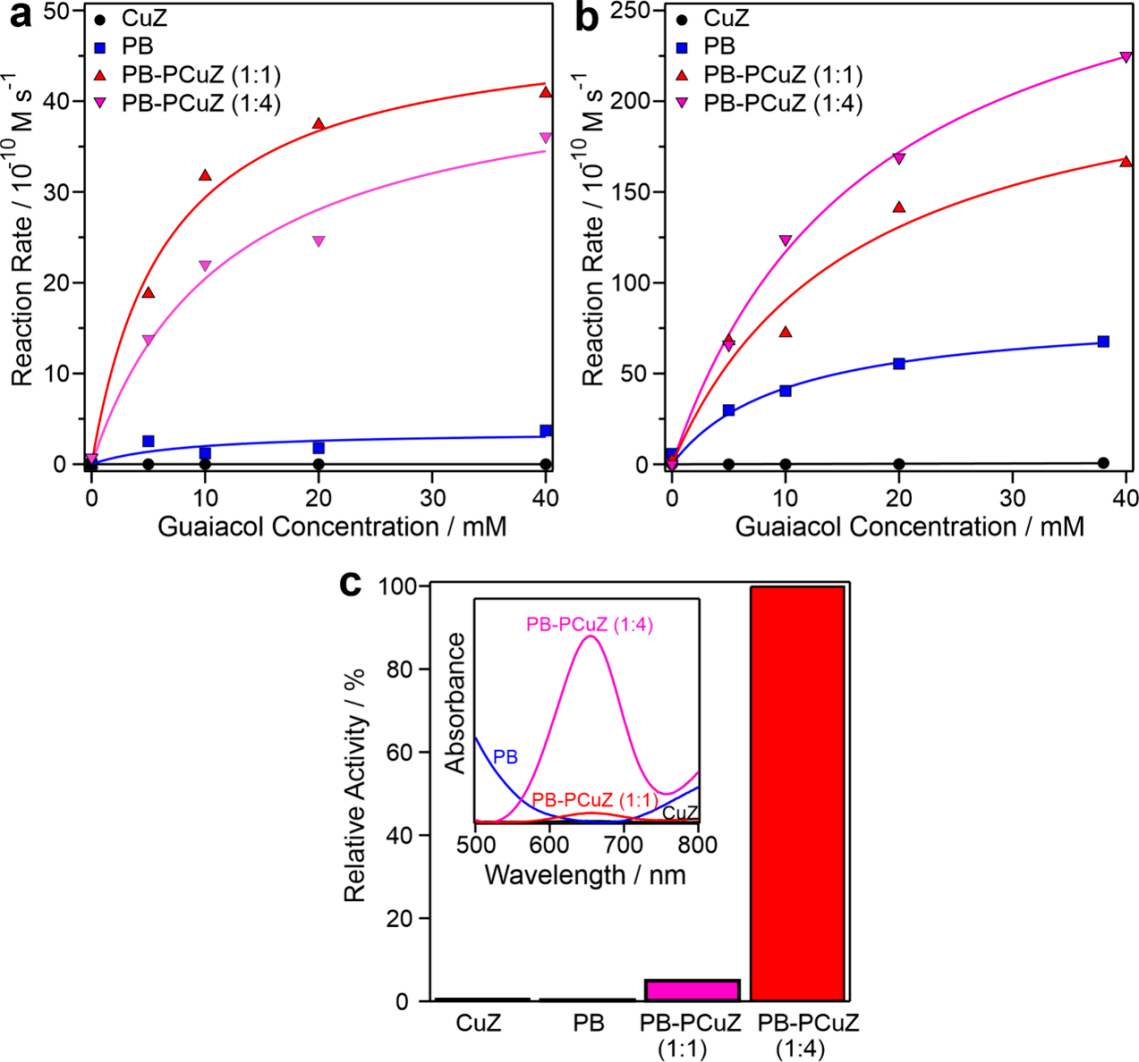
POD-like activity of the nanozymes and their mixtures with varying concentrations of guaiacol (a, b) and 5 mM TMB substrate (c). The lines in (a) and (b) represent the Michaelis–Menten fits with eq 5. The inset in (c) shows the UV–visible spectra taken after 10 min reaction time. The concentration of PB was maintained at 10 ppm in (a) and 40 ppm in (b) and (c) for PB-containing nanozymes. The CuZ data refer to 10 and 40 ppm of CuZ content in (a) and in (b) and (c), respectively.

The product of the SOD reaction, hydrogen peroxide, can be further converted by POD-mimicking compounds to water and molecular oxygen. The POD function of the present systems was evaluated by their ability to catalyze the oxidation of guaiacol and TMB in the presence of hydrogen peroxide (Figure 5). After monitoring the formation of the brown-colored tetraguaiacol product with UV–visible spectrophotometry, reaction rate data were extracted, and a Michaelis–Menten fit was performed (Equation 5). The experimental and calculated data are shown in Figure 5a,b. It was found that the CuZ itself does not possess POD-like activity. In contrast, PB is moderately POD active (Table S3), but its activity is significantly below other PB compounds reported earlier.^28^ Similar to the SOD assay results, the hindered POD activity of the PB nanocubes can also be attributed to the presence of the PVP coating.

At 10 ppm concentration (Figure 5a), CuZ shows no activity, while the POD-like function of PB is limited. However, the activity is significantly increased in the PB–PCuZ samples with maximum rate values and catalytic turnover rates increasing by more than an order of magnitude (Table S3), highlighting a coamplification of the PB POD-like activity by the CuZ present in the cocktails. The 1:1 ratio was found to be slightly more active than the 1:4 ratio in terms of maximum rates, while the Michaelis constant remained in the same range.

The activity of PB at 40 ppm (Figure 5b) has also increased upon mixing with PCuZ, but the synergistic effect was smaller; i.e., the POD-like activities of the PB–PCuZ cocktails were closer to the activity of PB. It is important to note that while at 10 ppm PB concentration the maximum reaction rate is similar for the 1:1 and 1:4 mass ratios, at 40 ppm the activity of PB–PCuZ systems increases with the PCuZ mass ratio. In the PB–PCuZ (1:4) system the CuZ concentration is high, 160 ppm. This increases the number of POD-active sites in the material, leading to an amplified POD-like activity, reflected in the elevated *v*_max_ and *k*_cat_ values, indicating that CuZ plays an active part in synergistic catalysis. Moreover, it was found that the mixing time does not influence the POD-like function significantly, since the activity of the freshly mixed cocktail was within the experimental error compared to the one after 30 min mixing time (Figure S8).

Further experiments were performed with the TMB substrate to confirm the above findings. The samples contained 0.5 mM TMB and 15 mM H_2_O_2_ apart from the nanozymes (Figure 5c). Neither CuZ nor PB showed significant POD-like activity with TMB, while the PB–PCuZ mixtures both exhibited POD-like functions, with the PB–PCuZ (1:4) being 20× more active than PB–PCuZ (1:1). This trend is in line with that obtained with the guaiacol substrate.

These results clearly point out that mixing PB nanozymes with PCuZ vastly improves the POD-like activity of the systems compared to the individual nanozymes and highlight the importance of accessibility of the active sites for the substrate. In the PB–PCuZ mixtures, the zeolite acts as cocatalyst, since it has no POD-like catalytic activity alone, but the zeolite surface is suitable for binding the guaiacol moieties as well as, owing to the PDADMAC pretreatment, the oppositely charged PB nanocubes. As neither the substrate nor PB can penetrate into the pores of the ZSM-5 structure due to steric hindrance,^60^ the improvement in the reaction rate is expected to be tied to the coadsorption of these materials on the zeolite surface. This creates an optimal reaction environment where the hindered activity of the polymer-coated PB nanocubes is counteracted by the increased local concentration of the substrate at the interface.

POD-like activities of similar materials were collected from the literature for comparison (Table S4). Remarkably, the PB–PCuZ systems outperform other PB materials with regard to SOD activity and possess comparable POD function to other PB and zeolite nanozymes. These findings indicate the benefits of these antioxidant nanozyme cocktails for broad-spectrum cascade scavenging of ROS.

### 3.5 Mechanism of Antioxidant Function

**Figure 6:**
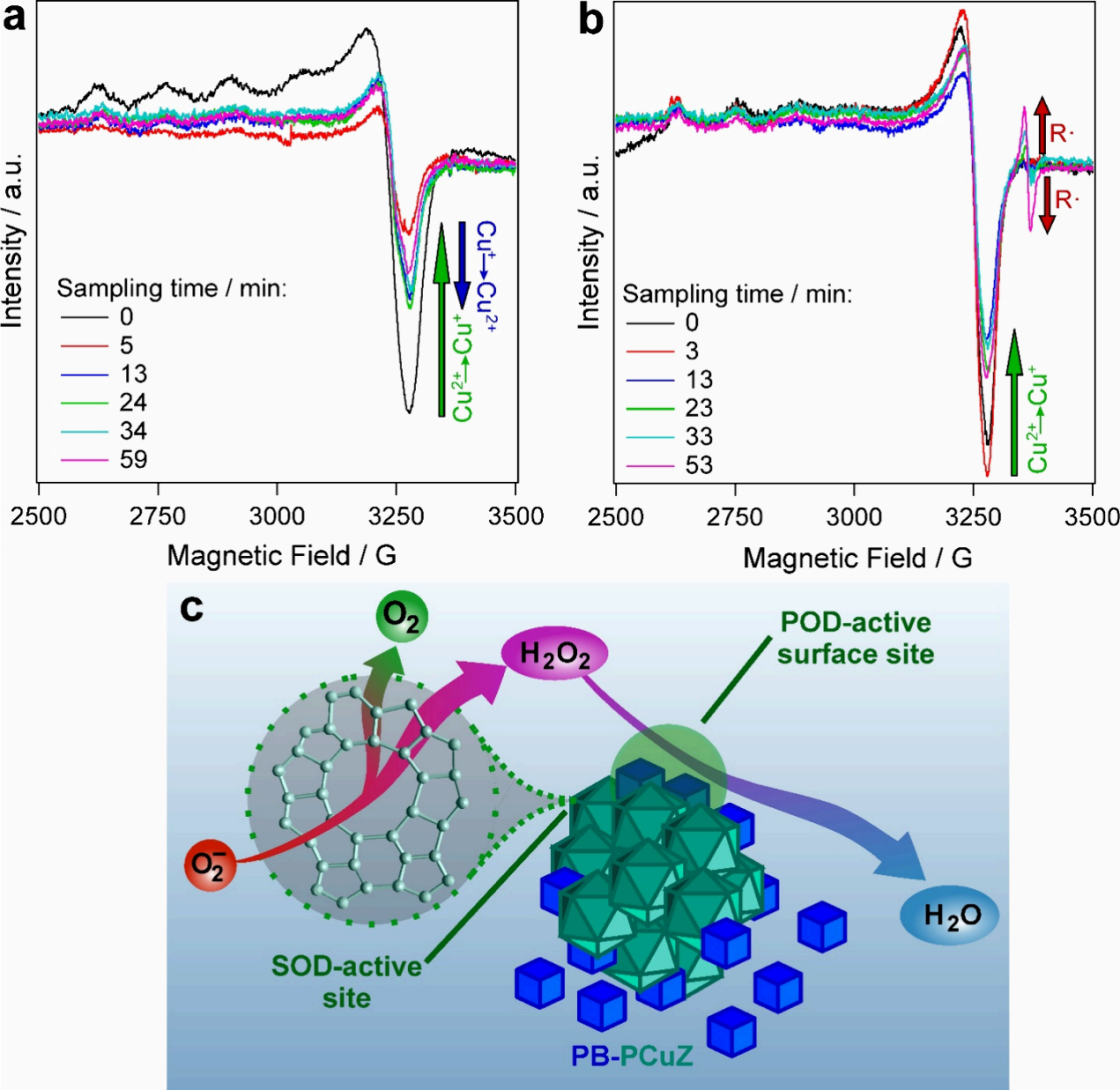
EPR spectra obtained at different reaction times in the SOD (a) and POD (b) assays. Schematic representation of the SOD/POD biocatalytic cascade based on EPR results (c). The CuZ was applied in (a) and PB–PCuZ (1:4) in (b). The CuZ concentration was kept constant at 200 ppm in the experiments. Guaiacol was used as substrate in (b). The intensity scale is identical in (a) and (b).

In light of the SOD and POD assay results, EPR studies were conducted to further explore the coamplified activity in the PB–PCuZ cocktails. In these experiments, the change of the characteristic signal of the copper(II) moieties between 2500 and 3500 G (see also Figure S9 for the ZSM-5 and PCuZ EPR spectra) was monitored. Modified versions of the assays were used to increase the PCuZ concentration to the point where it could be detected by EPR. Results of these experiments are shown in Figure 6.

To follow the SOD-like function (Figure 6a), CuZ was applied as the simplest highly SOD-active system in this study, while for the POD assay (Figure 6b) the use of PB–PCuZ (1:4) was necessary, as CuZ only acts as cocatalyst in this reaction. In each case, the reaction time was increased to 1 h. A significant decrease in the copper(II) signal can be observed during the SOD test within the first 3 min of the reaction, marked with the green arrow in Figure 6a. This is in line with the mechanism of the SOD function, where copper(II) is first reduced to copper(I) by the superoxide radical anions, and hence, the copper(II) EPR signal should decrease. Thereafter, the copper(I) ions are reoxidized (blue arrow) by the radicals reaching an equilibrium within the first 20 min. These results indicate that the CuZ operates according to the same mechanism as the native enzyme, which was not confirmed experimentally for SOD-mimicking materials earlier.

While the PCuZ was only expected to act as cocatalyst in the POD step of the cascade (Figure 6b), a decrease of the copper(II) signal can also be observed in the first 20 min of this experiment (green arrow). This indicates that, while CuZ is not POD-active by itself, the copper centers in the material still take an active part in the coamplified POD catalysis. At the same time, the evolution of a radical-associated signal occurs (red arrows) with a *g* value (dimensionless constant characteristic of a paramagnetic moiety’s response to the magnetic field; see also Table S5 for simulated anisotropic EPR parameters) of 2.0027, centered around 3365 G. This signal is wide (10 G), indicating a species, presumably the guaiacol dimer radical intermediate,^61^ bound to a metal center, which can be either the iron in PB or the copper or aluminum in CuZ. Note that a similar radical signal is not present in the SOD test (Figure 6a) as the superoxide radical anion produced in the xanthine–xanthine oxidase reaction is immediately converted to hydrogen peroxide by the nanozyme.

These experimental results imply that SOD-like catalysis can occur inside the CuZ framework, while the POD reaction involves both CuZ and PB and, thus, the latter process requires surface sites where the reaction partners are jointly present (Figure 6c).

## 4 Conclusions

This study reports the development of antioxidant nanozyme cocktails consisting of polyelectrolyte-coated zeolite particles (PCuZ) and polymer-templated PB nanocubes to mimic enzyme cascades. The polymer coatings inferred colloidal stabilization to the individual materials, rendering especially the PB dispersions to be processable in a wide range of experimental conditions. Charge reversal by polyelectrolyte adsorption allowed tuning of the surface charge of CuZ to prepare PB–PCuZ colloids. In the nanozyme cocktails designed, significant amounts of the PB particles were not adsorbed on the PCuZ surface but were also dispersed individually in the solution. In this way, highly SOD- and POD-active catalytic sites were created, facilitating a cascade that turns superoxide radical anions to water and molecular oxygen. The partitioning between the PCuZ interface and the bulk led to vastly improved POD-like activities compared to either component, while the SOD activity of the PCuZ is essentially unaffected by the presence of the less active PB. The EPR studies indicated that the porous structure of CuZ is beneficial for the interpenetration of ROS into the bulk material conferring high SOD activity. POD-active surface sites were also formed in the PB–PCuZ composites, utilizing the copper moiety of CuZ to coamplify the POD-like activity of PB. The PB–PCuZ cocktails outperform other PB-based SOD-active materials while also presenting comparable POD-like activities to similar nanozymes. This research marks a step forward in the application of nanozyme cocktails, as the fabricated PB–PCuZ mixture can mimic an enzymatic cascade turning the superoxide radical anion first into hydrogen peroxide via SOD-like function and then into water via POD-like operation. This report unambiguously confirms that coamplified antioxidant activity is possible between two enzyme mimicking materials in dispersions.

## Supporting Information Available

Normalized scattered intensities; Raman spectra and peak assignments; EPR parameters and spectra; POD activity data; literature data on SOD- and POD-like activities of PB and zeolite nanozymes; electrophoretic mobility and hydrodynamic radius data; PB dispersion images; EDX spectra and analysis results; UV–visible spectra; XPS spectra and analysis results (PDF)
