# Supplementary material for: Coamplified Nanozyme Cocktails for Cascade Reaction-Driven Antioxidant Treatments
Source: ACS Appl Mater Interfaces. 2024 Sep 24;16(40):54486–96. doi: 10.1021/acsami.4c12511 (PMC11472261; doi:10.1021/acsami.4c12511)
Supplement: Supplementary file 1 — am4c12511_si_001.pdf [file am4c12511_si_001.pdf]

Supporting Information (SI)

Co-Amplified Nanozyme Cocktails for Cascade Reaction-Driven Antioxidant Treatments

Tibor G. Halmagyi,[‡] *Attila Voros,*[‡] *Szilard Saringer,*[‡] *Viktoria Hornok,*[‡] *Nora V. May,*[†] *Gergely F. Samu,*[ǂ] *Imre Szenti,*[§] *Adel Szerlauth,*[‡] *Zoltan Konya,*[§] *and Istvan Szilagyi**[‡]

[‡]MTA-SZTE Momentum Biocolloids Research Group, Department of Physical Chemistry and Materials Science, Interdisciplinary Centre of Excellence, University of Szeged, 1 Rerrich Béla tér, 6720 Szeged, Hungary

[†]Centre for Structural Sciences, HUN-REN Research Centre for Natural Sciences, 2 Magyar tudósok körútja, 1117 Budapest, Hungary

[ǂ]Department of Molecular and Analytical Chemistry, University of Szeged, 7 Dóm tér, 6720 Szeged, Hungary

[§]Department of Applied and Environmental Chemistry, University of Szeged, 1 Rerrich Béla tér, 6720 Szeged, Hungary

*Corresponding author. Email: szistvan@chem.u-szeged.hu

Characterization of the CuZ Material. Analysis of the composition and structure of the CuZ sample was carried out by XPS and EPR. First, the copper oxidation state distribution on the surface of the particles was explored upon ion exchange of ZSM-5 (Figure S7a). Two different copper environments could be identified from the measurement results. Aside from the expected copper(II) satellites at 934 eV binding energy (Figure S7b), metallic copper can also be located at 932.8 eV.[1] The copper(II) ion-to-metallic copper ratio was 78.5-to-21.5 %. The copper content of CuZ (1.5 % total) is comparable with that determined with SEM-EDX (0.93 %), indicating that the copper(II) ion exchange progressed in the bulk of the zeolite material beyond the surface.

In addition, EPR measurements were performed on the ZSM-5 and CuZ samples at 77 K in water-methanol (4:1) mixture in the 2300-3800 G regime, which is characteristic of copper(II) moieties (Figure S8a). The ZSM-5 did not contain any copper, so no signals were identified for this sample. CuZ exhibited signals characteristic for copper ion-exchanged zeolites,[2] with a symmetric, elongated octahedral geometry, confirming the successful ion exchange and the presence of copper(II) ions inside the zeolite structure. In addition, simulation of EPR spectra of CuZ was performed at two concentrations (400 and 40 ppm, Figure S8b). The anisotropic EPR parameters were determined from the simulation (Table S5).

Table S1. Normalized scattered intensities of the individual nanozyme components as measured with 3DLS at 40 ppm material concentration and pH 4. The scattering angle was 150°.

Material	Normalized scattered intensity / kHz/mW
CuZ	0.61 ± 0.35
PB	14.43 ± 0.11

Table S2. Peak assignments of Raman spectra shown in Figure S3.

CuZ	
Raman Shift (cm^{-1})	Assignment
295	δ(T-O-T)
380	ν_s(T-O-T)
400-500	ν(Si-O-Si), δ(O-Si-O(Al)), δ(Si-O)
790	ν_s(Si-O-Si)
PB	
Raman Shift (cm^{-1})	Assignment
279	δ(Fe-CN-Fe)
450-620	ν(Fe-C)
2095	ν(Fe^{II}-CN-Fe^{III})
2156	ν(C≡N)
PB-CuZ	
Raman Shift (cm^{-1})	Assignment
279	δ(Fe-CN-Fe)
450-620	ν(Fe-C)
2096	ν(Fe^{II}-CN-Fe^{III})
2157	ν(C≡N)

ν: stretching vibration, ν_s: symmetrical stretching vibration, δ: bending vibration

Table S3. Summary of the POD-like activity of the dispersions studied, compared to the horseradish peroxidase (HRP) enzyme. Guaiacol substrate was used in each case.

Nanozyme	v_{max} / 10^{-10} M s^{-1}	K_M / mM	k_{cat} / s^{-1}
PB	3.7 ± 1.9[a]<br>84.7 ± 7.9[b]	8.3 ± 1.3[a]<br>10.2 ± 2.6[b]	66.7 ± 34.3[a]<br>381.9 ± 35.6[b]
PB-PCuZ (1:1)	48.9 ± 3.2[a]<br>236.5 ± 41.9[b]	6.6 ± 1.5[a]<br>16.2 ± 6.6[b]	881.8 ± 57.7[a]<br>1066.2 ± 188.9[b]
PB-PCuZ (1:4)	44.8 ± 5.6[a]<br>323.7 ± 17.4[b]	11.9 ± 5.6[a]<br>17.7 ± 2.1[b]	807.9 ± 101.0[a]<br>1459.4 ± 78.4[b]
HRP	2800[c]	3.23[c]	224.0[c]

[a]With 10 ppm PB concentration; [b]With 40 ppm PB concentration; [c]Values taken from reference (3).

Table S4. SOD and POD activity results of various PB and zeolite-based nanozymes.

Nanozyme	SOD	POD		Reference
	IC_{50} / ppm	v_{max} / 10^{-10} M s^{-1}	K_M / mM	
PB	1.64	67.1[a]	2.19[a]	4
PB	33.7	637.8[b]	7.825[b]	5
PB	–	1737[b]	4.729[b]	6
Co-PB	–	550[b]	3.594[b]	
Ni-PB	–	14[b]	1.041[b]	
Cu-PB	–	37[b]	2.063[b]	
PB	–	633[d]	0.68[d]	7
Mn-PB	–	350[d]	0.8[d]	
Nano-Y/Cu	–	221[b]	0.54[b]	8
Nano-Y/Ag/Cu/Zn	–	876[b]	0.076[b]	9

[a]Guaiacol substrate; [b]Hydrogen peroxide substrate; [c]3,3',5,5'-tetramethylbenzidine substrate. [d]2,2'-azino-bis(3-ethylbenzothiazoline-6-sulfonic acid) substrate.

Table S5. Anisotropic EPR parameters of components obtained in the simulation of the EPR spectra. The experimental errors were ±0.002 for g and ± 2 G for A values.

Parameter	$g_{\perp}$	$g_{\parallel}$	$A_{\perp}$(G)	$A_{\parallel}$(G)	$g_{0,calc}$
Value	2.086	2.384	10	126	2.185

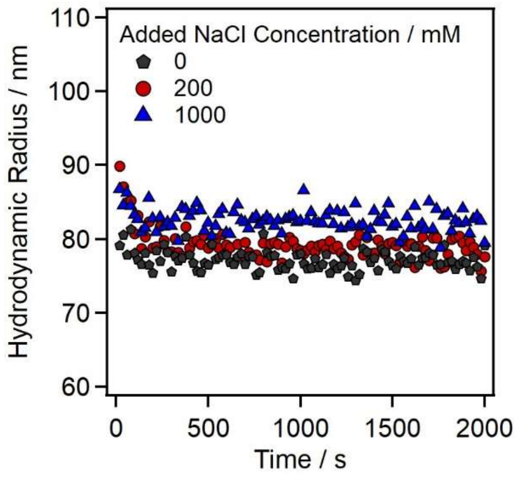

Figure S1. Hydrodynamic radii of PB particles (40 ppm) as a function of time at different concentrations of NaCl electrolyte at pH 4.

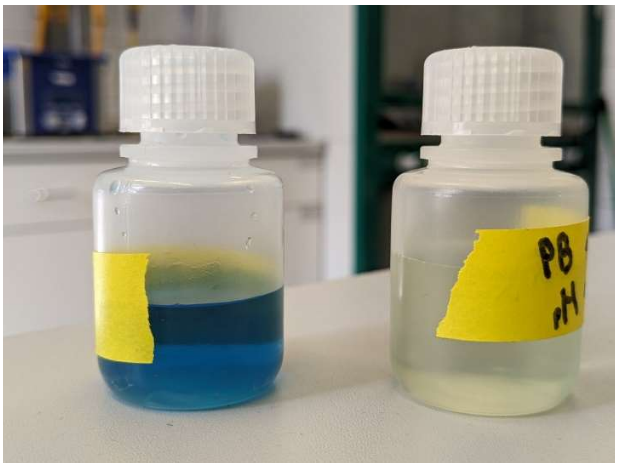

Figure S2. Dispersions of 40 ppm PB at pH 10 immediately after mixing (left) and after one week (right). The decolorization may indicicate slow dissolution of the particles.

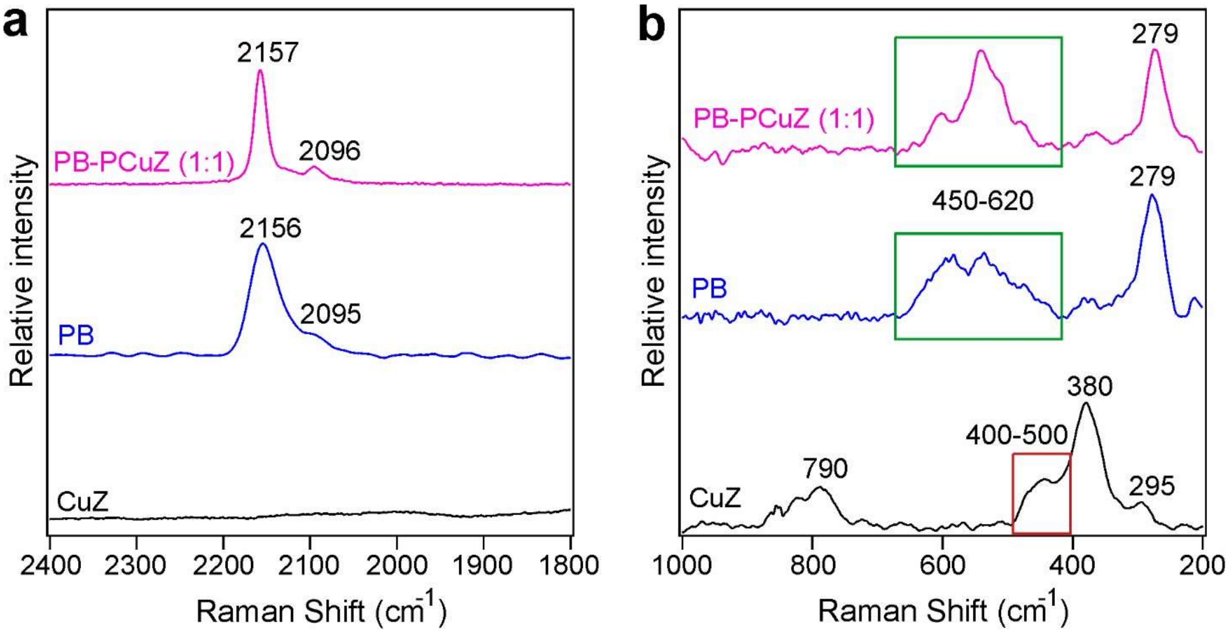

Figure S3. Raman spectra of the PB, CuZ and PB-PCuZ (1:1) nanozymes in the high- (a) and low-wavenumber regions (b).

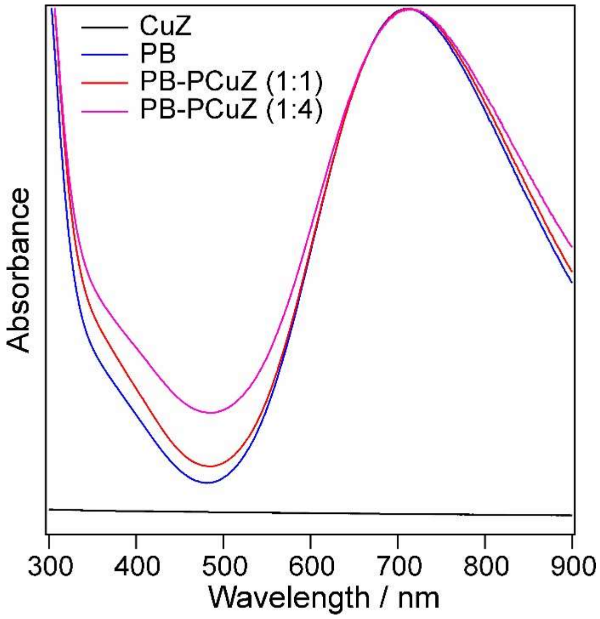

Figure S4. UV-visible spectra of PB, CuZ and PB-PCuZ nanozymes at 40 ppm PB concentration (PB, PB-PCuZ) or at 40 ppm CuZ concentration (CuZ) at pH 4 and 0.1 mM ionic strength.

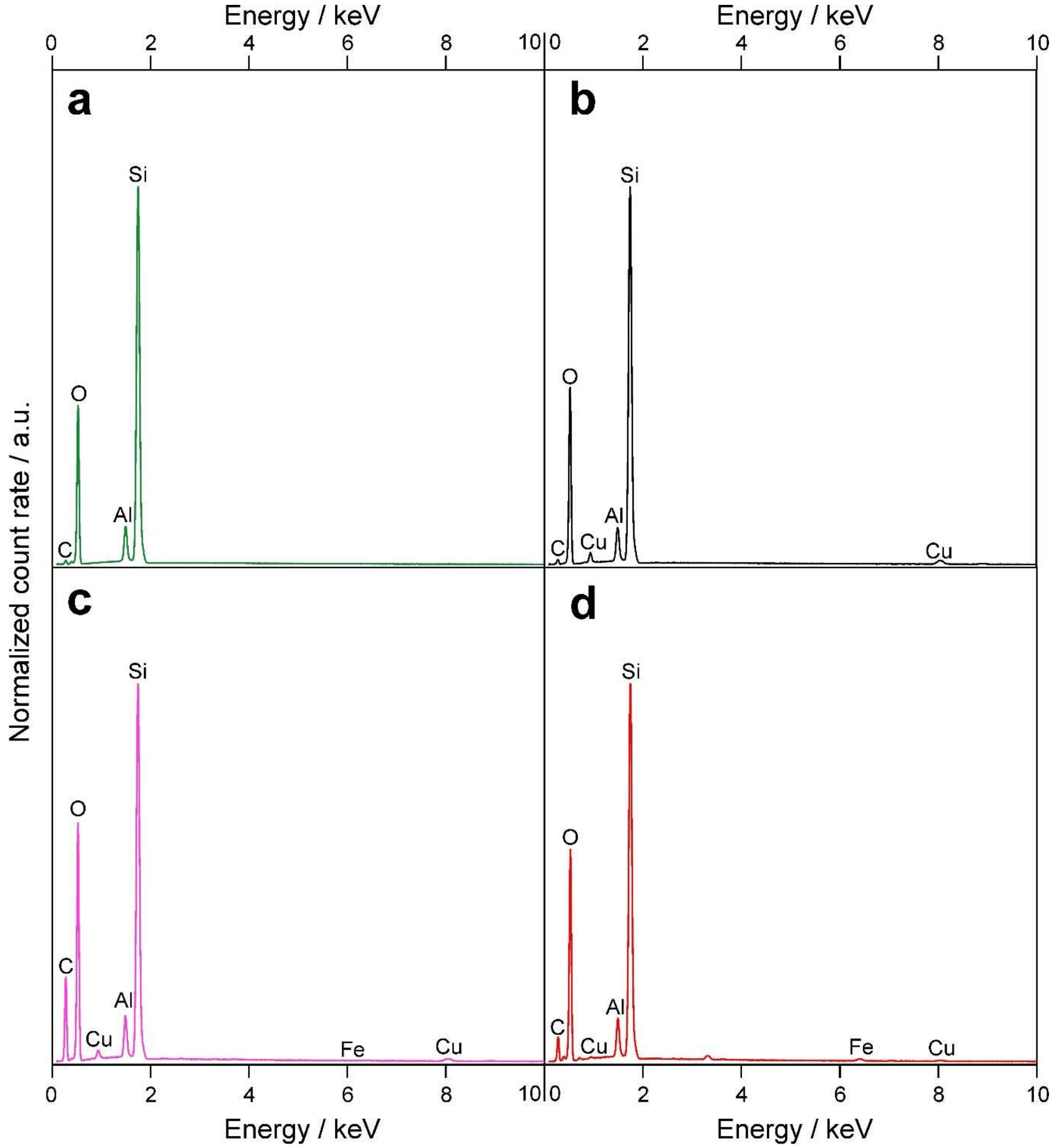

Figure S5. SEM-EDX spectra of ZSM-5 (a), CuZ (b), PB-PCuZ (1:4) (c) and PB-PCuZ (1:1) (d).

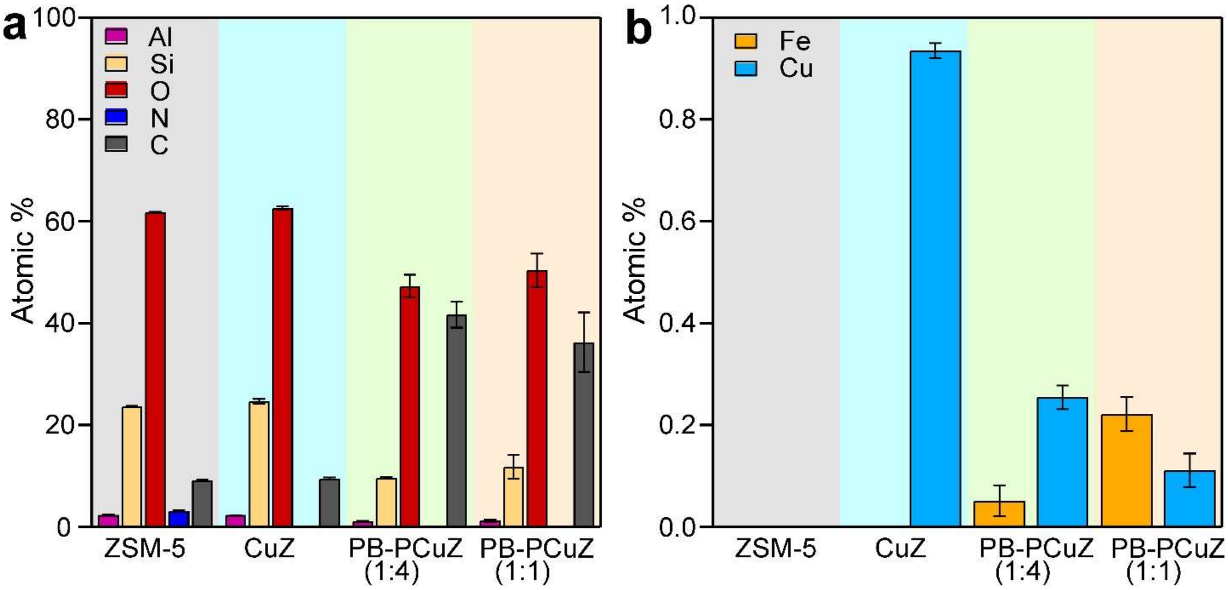

Figure S6. Results of SEM-EDX measurements on nanozyme samples. Major elements shown in (a), while trace elements in (b). Error bars represent deviation from average values acquired in 3 locations of the same sample.

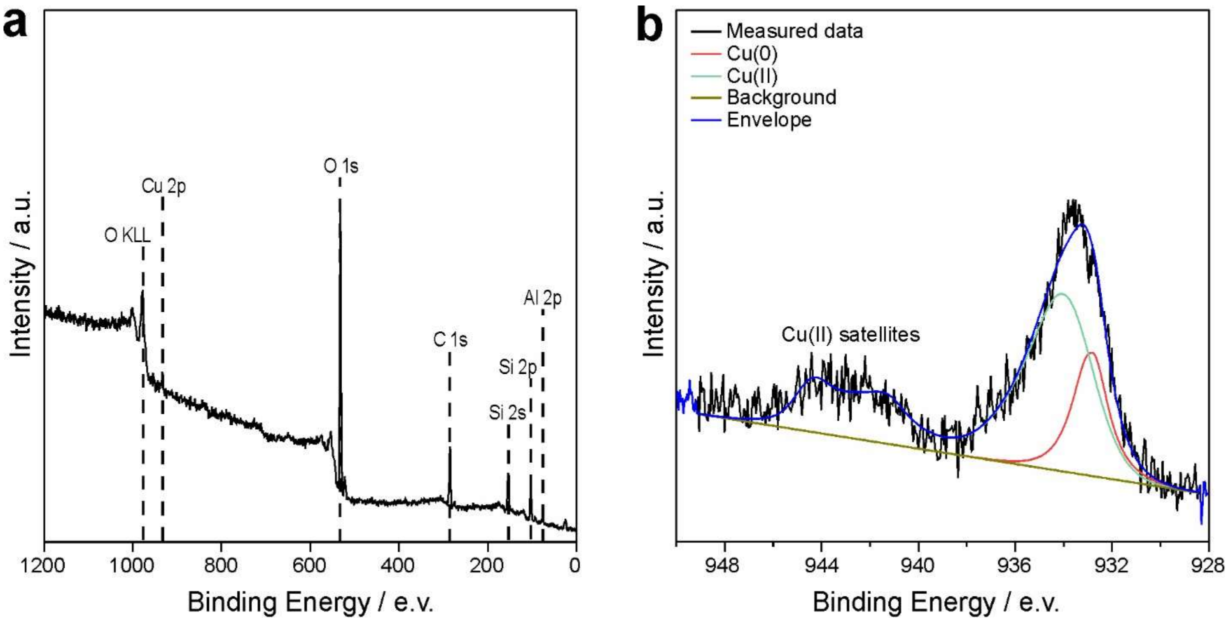

Figure S7. XPS spectrum of the CuZ sample (a) and evaluation of the Cu $2p_{3/2}$ region (b).

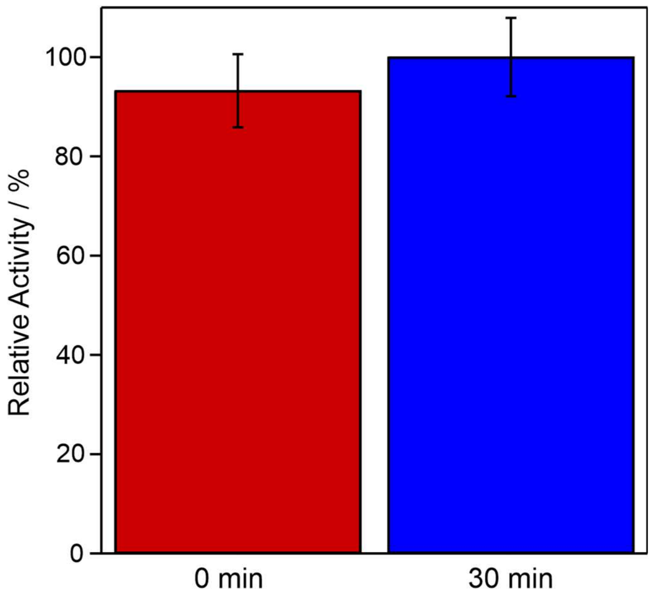

Figure S8. Relative POD-like activity of the PB-PCuZ cocktails used immediately after mixing and after a 30 minute mixing time. Guaiacol (40 mM) was used as substrate in the experiments.

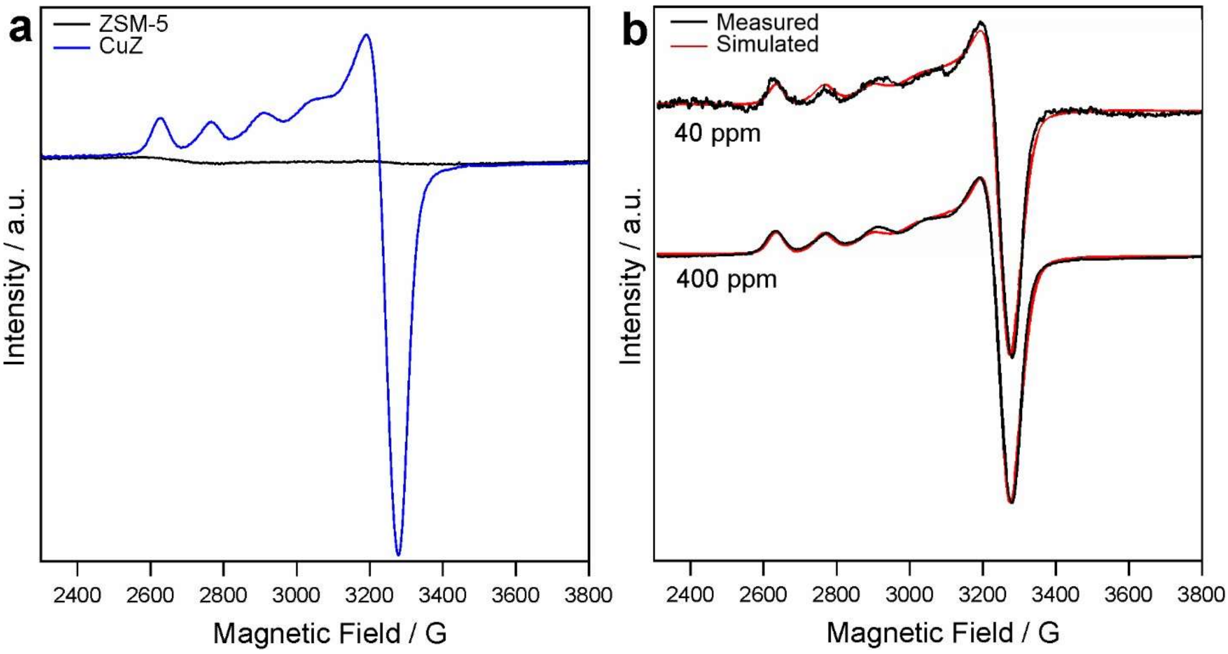

Figure S9. Electron paramagnetic resonance spectra of 400 ppm PCuZ samples (a) and comparison of simulated fits and normalized PCuZ spectra at different concentrations (b). The measurements were performed at 77 K in water-methanol (4:1) mixtures.

REFERENCES

(1) Biesinger, M. C. Advanced analysis of copper X-ray photoelectron spectra. *Surf. Interface Anal.* **2017**, *49*, 1325-1334.

(2) Carl, P. J.; Larsen, S. C. EPR study of copper-exchanged zeolites: Effects of correlated *g*- and *A*-strain, Si/Al ratio, and parent zeolite. *J. Phys. Chem. B* **2000**, *104*, 6568-6575.

(3) Pavlovic, M.; Rouster, P.; Somosi, Z.; Szilagyi, I. Horseradish peroxidase-nanoclay hybrid particles of high functional and colloidal stability. *J. Colloid Interface Sci.* **2018**, *524*, 114-121.

(4) Alsharif, N. B.; Samu, G. F.; Sáringer, S.; Muráth, S.; Szilagyi, I. A colloid approach to decorate latex particles with Prussian blue nanozymes. *J. Mol. Liq.* **2020**, *309*, 113066.

(5) Xie, X.; Zhao, J.; Gao, W.; Chen, J.; Hu, B.; Cai, X.; Zheng, Y. Prussian blue nanozyme-mediated nanoscavenger ameliorates acute pancreatitis via inhibiting TLRs/NF-κB signaling pathway. **2021**, *11*, 3213-3228.

(6) Chen, J. X.; Wang, Q. Q.; Huang, L.; Zhang, H.; Rong, K.; Zhang, H.; Dong, S. J. Prussian Blue with intrinsic heme-like structure as peroxidase mimic. *Nano Res.* **2018**, *11*, 4905-4913.

(7) Zhang, Y. N.; Kudriashov, D.; Pershina, L.; Offenhäusser, A.; Mourzina, Y. Intrinsic multienzyme-like activities of the nanoparticles of Mn and Fe cyano-bridged assemblies. *Nanomaterials* **2022**, *12*, 2095.

(8) Moradpour, Z.; Kohnehshahri, M. H.; Shekarloo, M. V.; Jalili, V.; Zendehdel, R. Peroxidase-like reaction by a synergistic inorganic catalyst colloid: a new method for hydrogen peroxide detecting in air samples. *Colloid Polym. Sci.* **2021**, *299*, 1567-1575.

(9) Ravannakhjavani, F.; Dehghan, S. F.; Panahi, D.; Moradpour, Z.; Zendehdel, R. Multi-metal nanozyme properties for colorimetric peroxidase reaction: Overview of an applicable method validation for H_2O_2 detection. *J. Inorg. Organomet. Polym. Mater.* **2024**, *34*, 818-826.
